# Broad thermal spectrum metagenomic laccase with action for dye decolorization and fentin hydroxide treatment

**DOI:** 10.1186/s13568-022-01375-0

**Published:** 2022-03-23

**Authors:** Natália Sarmanho Monteiro Lima, Elisângela Soares Gomes-Pepe, João Carlos Campanharo, Eliana Gertrudes de Macedo Lemos

**Affiliations:** 1 Department of Agricultural and Environmental Biotechnology, São Paulo State University, Jaboticabal Campus, Jaboticabal, São Paulo State 14884-900 Brazil; 2 Molecular Biology Laboratory, Institute for Research in Bioenergy (IPBEN), Jaboticabal, São Paulo State 14884-900 Brazil; 3 Graduate Program in Agricultural and Livestock Microbiology, School of Agricultural and Veterinarian Sciences, São Paulo State University (UNESP), Jaboticabal, SP Brazil

**Keywords:** Laccase, Metagenomics, Dyes, Bioremediation

## Abstract

Laccases are multicopper oxidases that act on various phenolic and non-phenolic compounds, enabling numerous applications including xenobiotic bioremediation, biofuel production, drug development, and cosmetic production, and they can be used as additives in the textile and food industries. This wide range of uses makes these enzymes extremely attractive for novel biotechnology applications. Here, we undertook the kinetic characterization of LacMeta, a predicted as homotrimeric (~ 107,93 kDa) small laccase, and demonstrated that this enzyme performs best at an acidic pH (pH 3–5) towards ABTS as substrate and has a broad thermal spectrum (10–60 °C), which can promote high plastic action potential through dynamic environmental temperature fluctuations. This enzyme showed following kinetic parameters: k_cat_ = 6.377 s^−1^ ± 0.303, K_m_ = 4.219 mM, and V_max_ = 24.43 µM/min (against ABTS as substrate). LacMeta almost completely degraded malachite green (50 mg/mL) in only 2 h. Moreover, the enzyme was able to degrade seven dyes from four distinct classes and it respectively achieved 85% and 83% decolorization of methylene blue and trypan blue with ABTS as the mediator. In addition, LacMeta showed potential for the degradation of two thirds of an agricultural fungicide: fentin hydroxide, thus demonstrating its biotechnological aptitude for bioremediation. The results of this study suggest that LacMeta has potential in textile wastewater treatment and that it could help in the bioremediation of other human/environmental toxins such as pesticides and antibiotic compounds belonging to the same chemical classes as the degraded dyes.

**Graphical Abstract:**

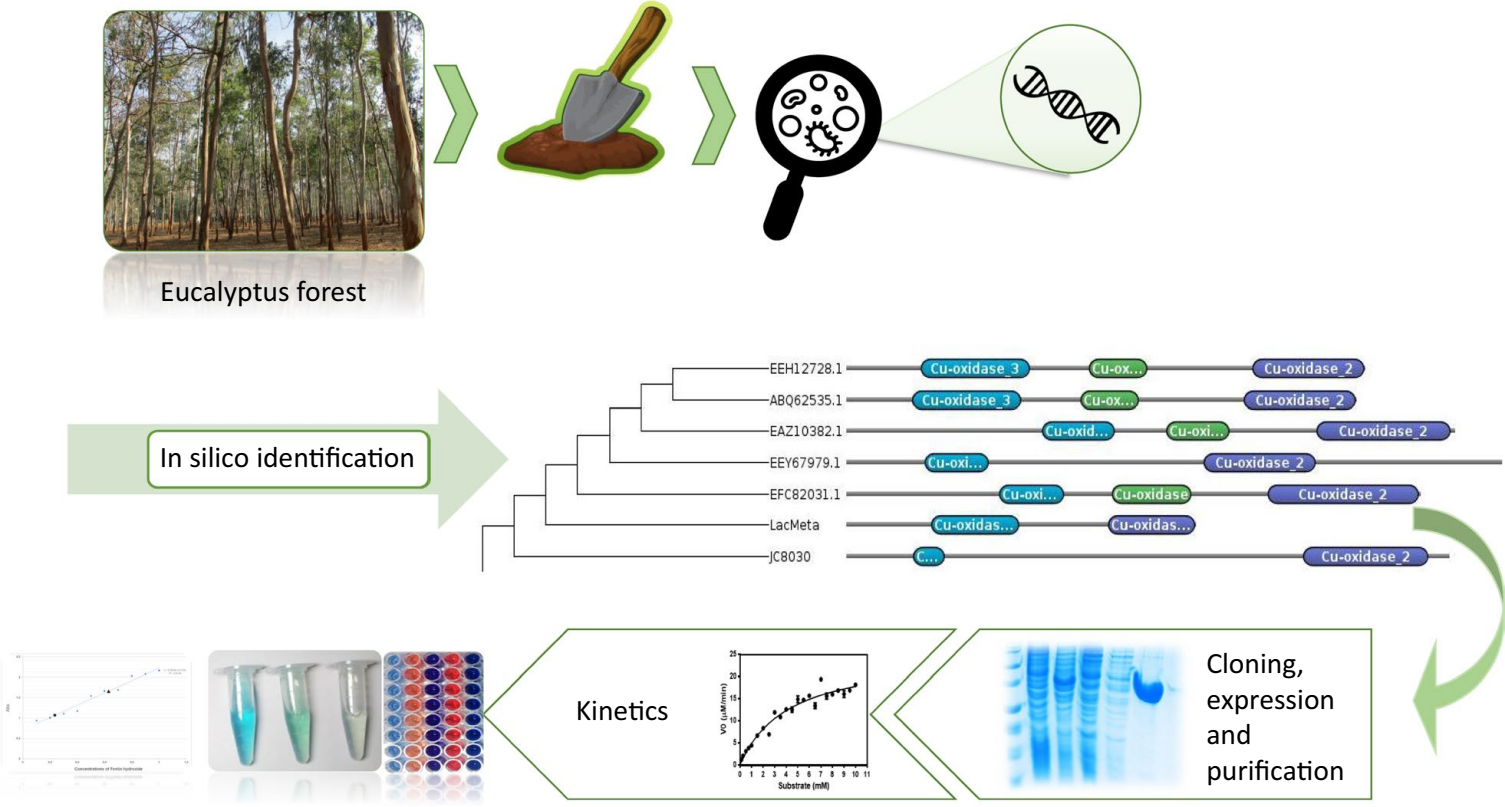

**Supplementary Information:**

The online version contains supplementary material available at 10.1186/s13568-022-01375-0.

## Key points

- LacMeta is a new two-domain laccase with activity over a wide temperature range
- LacMeta maintained 50% activity after 5 months of storage at 4 °C.
- Laccase was able to degrade in 2 h the Malachite Green dye, and had the potential to degrade fentin hydroxide

**Supplementary Information:**

The online version contains supplementary material available at 10.1186/s13568-022-01375-0.

## Introduction

The global production of dyes can reach up to 800,000 tons per year, with approximately 10 to 15% of this production lost to industrial processes, aggravating water pollution worldwide (Hassaan and Nemr 2017). This worldwide problem is particularly pronounced in Brazil, where the textile industry is the second largest sector in the country, surpassed only by the food and beverage industry, with an average production of 1.3 million tons. Within Brazil, São Paulo state is the largest single manufacturing site, accounting for almost 30% of the national production. Textile dyes are predominantly organic compounds with complex aromatic structures, making them difficult to degrade using xenobiotic approaches (Afreen et al. 2017). This means that textile effluents often retain high concentrations of these dyes, which form a layer on the water surface, which reduces the amount of dissolved oxygen and restricts light penetration, thereby harming the photosynthetic organisms present in this aquatic environment, which can have devastating consequences for the entire ecosystem (Ali 2010). The lack of information regarding the correct use of dyes, including the effects of their uncontrolled use and the incorrect disposal of wastewater into rivers can damage human health and compromise the ecological balance (Mathur et al. 2006). For example, when some azo dyes undergo dissociation in water, they produce carcinogenic, mutagenic, and allergenic compounds that can have a significant impact on the environment (Chung 2016). Some microorganisms have the ability to bioremediate these dyes; for example, *Trametes sanguineus* (Balcázar-López et al. 2016), *Lysinibacillus sphaericus* (Chantarasiri and Boontanom 2017), and *Acinetobacter baumannii* (Kuppusamy et al. 2017) all produce enzymes that facilitate organic compound dissociation. Other compounds commonly found in wastewater are pesticides used in agriculture to protect and prevent pests, increase productivity, and thereby increase economic gain. They have a high chemical diversity, can be organic or inorganic, and act as herbicides, fungicides, insecticides, nematicides, and fumigants (Verma et al. 2014). Incorrect application and improper storage of pesticides are some of the factors that lead to contamination of soil and surface and groundwater, as pesticides are highly stable and soluble, and they may remain in the environment for long periods (Struthers et al. 1998; Narayanan et al. 2020). Given these observations, the search for new microorganisms and microbial enzymes that can be used in wastewater treatment has gained momentum, with some interesting results, although more studies are needed to develop efficient and cost-effective processes. Enzymatic treatment has the advantage of operation at low and high concentrations of the contaminant and performance at a wide range of pH, temperature, and salinity (Durán and Esposito 2000). Generally, bacterial enzymes are tolerant to a wider range of temperatures and pH, as well as to environmental stresses (Ulrich et al. 2008; Li et al. 2020). The search for new biomolecules has increased, and consequently, the search for new microorganisms has increased. Traditional search methods depend on the cultivation of these microorganisms; however, metagenomics has facilitated the search for new molecules and access to detailed information that could not be achieved before, thereby reducing the dependence on cultivation.

Laccase enzymes [(benzenediol: oxygen reductases (EC 1.10.3.2)] are part of a family of multicopper oxidases (MCOs), which include ferroxidases (EC 1.16.3.1) and ascorbate oxidases (EC 1.10.3.3). MCO enzymes have been shown to have several distinct functions, including copper/iron transport metabolism (ferroxidases) to polyphenol oxidation (laccase) (Copete et al. 2015). MCOs are characterized by their four redox-active cupric ions, which are generally organized into three domains: T1, T2, and T3 (Brander et al. 2014). Laccases are oxidative enzymes that act on various polyphenolic, phenolic, and non-phenolic compounds, with some enzymes demonstrating affinity for aromatic amines as well (Balcázar-López et al. 2016). This broad spectrum makes these enzymes suitable targets for several applications, including pulp delignification, bleaching, water bioremediation, various applications in the textile industry, beverage treatment, enzymatic and immunochemical assays (Balcázar-López et al. 2016), bioethanol production (Fang et al. 2015), and several unique applications in the pharmaceutical and cosmetic industries (Mate and Alcalde 2017). These enzymes can be found in vascularized plants, fungi, insects, and bacteria (Copete et al. 2015) as well as in mammals (including humans), mollusks, sea sponges, and octopuses (Janusz et al. 2017). Laccase-encoding genes have been identified in gram-positive and gram-negative bacteria, and even in bacteria that live in extreme environments, including *Thermus thermophilus*, which produces hyperthermophilic laccase (Piscitelli et al. 2010). Bacterial laccases are known to have several advantages over their fungal counterparts, including better stability under alkaline conditions (Sharma et al. 2007), high thermal/chemical stability, and salinity tolerance (Martins et al. 2015), making them prime candidates for industrial applications. Several enzymes with biotechnological applications have been described in several species of gram-positive bacteria, including several species belonging to *Actinobacteria*, particularly *Streptomyces*. These bacteria are known to produce a number of commercially valuable compounds, including antibiotics and various enzymes, and their capacity for enzyme production is attributed to their wide environmental and host ranges, which include deserts, Arctic glaciers, insects, plants, oceans, and soil (Endo 2003; Cheng et al. 2015). Characterized laccases from this genus, including those from *Streptomyces coelicolor* (Dubé et al. 2008), and *Streptomyces psammoticus* (Niladevi et al. 2008), have been shown to have several unique attributes, including having only two domains instead of the three characteristic domains and maintaining their activity via trimeric and/or quaternary structures. Due to their lack of a modular domain, these enzymes are referred to as “SLACs” (from the term “small laccases”) (Ausec et al. 2011). Functional studies of some of these SLACs have broadened the range of biotechnological applications of laccases. One example is the recombinant laccase (SilA) from *Streptomyces ipomoeae*, which was initially identified in a textile degradation study (Molina-Guijarro et al. 2009) and was shown to be an efficient degrader of ciprofloxacin (CIPRO) and norfloxacin (NOR), both of which are fluoroquinolones found frequently in contaminated aquatic environments because of their low biodegradability (Blánquez et al. 2016).

Considering the importance of addressing the water pollution problem inherent to textile manufacturing, it is critical that we continue to expand our repertoire and understanding of laccases. To this end, this study was designed to evaluate the heterologous expression and kinetic attributes of a novel metagenomic SLAC-like laccase, LacMeta. Here, we show that this enzyme can degrade seven different dyes from four different classes and one pesticide, making it a potentially useful enzyme for the treatment of textile waste and for reducing water pollution.

## Material and methods

### Laccase in silico analysis

The putative laccase enzyme was identified using homology analysis of metagenomic sequences retrieved using a sequence-driven cluster prospecting assay for class II polyketide antibiotics (PKS II), derived from the cosmid clone B5pl37 isolated from the eucalyptus soil metagenomic library (Gomes et al. 2013; Gomes-Pepe et al. 2016). In this cluster, several other enzymes were found, including LacMeta (ORF11) as can be seen in Fig. 10 of the work (Gomes et al. 2013). The degree of shared similarity between the predicted peptide sequence for this putative laccase, ORF13-B5pl37 (named *lacmeta*), and previously characterized enzymes found in the “National Center for Biotechnology Information” (NCBI collections “Non-redundant,” “UniProtKB/SwissProt,” and metagenomic proteins sequence) database were obtained using BLAST (“Basic Local Alignment Search Tool”). Multiple alignments were performed using Clustal Omega software (version 1.0.3). The possible presence of signal peptides was also investigated using the SignalP program (Dyrløv Bendtsen et al. 2004).

To determine the domain assembly and structure of these enzymes, sequences were evaluated using the DoMosaics program and analyzed based on sequence homology (Moore et al. 2014). The Pfam domain family comparison tool was integrated into the DoMosaics program and used to retrieve information about the laccase domains from sequence comparisons using the Hidden Markov Model. These domain sets were manually combined and used as individual entries to produce a single phenetic dendrogram. To complete this analysis, all the structures were subjected to a phenotypic alignment in Clustal Omega version 1.0.3 (Sievers et al. 2014), and then a dendrogram was constructed using the neighbor-joining method. The sequences used for these analyses were obtained from the MCO database “Laccase and Multicopper Oxidase Engineering Database,” LccED (https://lcced.biocatnet.de/), which is a cured multicopper oxidase database structured according to families used as source for standard sequences. Besides using it as a search tool, the database can also be used to identify sequences.

### Recombinant protein expression, extraction, and purification

The putative LacMeta gene was artificially synthesized, and the codon was optimized for expression in *Escherichia coli* (GenOne, http://www.genone.com.br/) and then cloned into pET-28a using *Eco*RI and *Hin*dIII (Novagen, Madison, WI, USA). After transformation, the single BL21 (DE3) colonies from transformed cells (positive for pET28a + lacmeta) were subjected to expression condition optimization to evaluate the optimal temperature, isopropyl-β-d thiogalactopyranoside (IPTG) concentration, and degree of agitation. After determining these conditions, the cells were again cultured in 50 mL Luria–Bertani (LB) medium containing 50 µg/mL kanamycin and shaken at 200 rpm at 37 °C for 16 h to obtain the pre-inoculum. Overexpression of the recombinant protein was then induced by adding 1% of the pre-inoculum to 1 L of LB medium (50 µg/mL kanamycin) and then cultured as described above until they reached an OD_600_ of 0.4–0.6, when 0.1 mM IPTG was added to induce protein expression. This induction was allowed to continue for 20 h at 30 °C with rotation at 200 rpm. After the incubation period, the cells were centrifuged and resuspended in 10 mL of lysis buffer (50 mM sodium phosphate pH 8.0, 200 mM NaCl, 10 mM imidazole, and 10% glycerol) and incubated with 4 µg/mL lysozyme for 1 h on ice. Cells were ultrasonically disrupted using a Branson Sonifier 250 sonicator (Branson, Connecticut, USA) at a cyclic rate of 20% with 10 cycles of 10 pulses at 10 s intervals. The disrupted cells were centrifuged for 30 min at 6387×*g* (Sorvall RC5C; Kendro Lab Products, Ashville, NC, USA) at 4 °C to obtain the extracts. The soluble extract was incubated for 1 h at 4 °C while shaking with 3.5 mL Ni^2+^–NTA resin (Qiagen, Hilden, Germany) previously equilibrated with sodium phosphate buffer containing 20 mM imidazole. After this incubation, the sample composed of the resin and bound extract was packed in a Poly Prep chromatography column (Bio-Rad, Hercules, CA, USA) and subjected to a 20 mM to 1 M imidazole gradient. The protein of interest was eluted using a buffer containing 500 mM imidazole.

This recombinant protein was then concentrated using an Amicon Ultra-15 filter with a molecular cut-off of 10 kDa (MWCO 10) (Merck Millipore, Billerica, MA, USA), and subjected to additional chromatography to remove contaminants and estimate the molecular weight and oligomeric state of these compounds. Molecular exclusion chromatography was conducted using AKTA pure (GE Healthcare Bio-Sciences, Uppsala, Sweden) and Hiload 16/600 Superdex column 200 column (GE Healthcare Bio-Sciences, Uppsala, Sweden) at a flow rate of 0.5 mL/min, injection of 2 mL, and pressure of 0.42 mPa. Flow, injection, and fractionation parameters were controlled using UNICORN 5.0 software (Amersham Bioscience Co., Piscataway, NJ). Protein concentration estimation in the form of an apoenzyme (not bound to copper ions) was performed using a spectrophotometer (Nanodrop ND-1000, Thermo Scientific, Waltham, MA, USA). The equipment was calibrated using the relevant protein extinction coefficient values, and their respective molecular mass values were estimated using the ProtParam software (http://web.expasy.org/protparam/).

### Copper binding and determination of recombinant protein (holoenzyme) concentration

The purified recombinant protein was incubated with 1 mM CuSO_4_ for 6 h to allow for the filling of the copper binding sites and thus the creation of the active holoenzyme bound to its co-factor (copper). After incubation, excess copper was removed using 20 mM sodium phosphate buffer pH 7 supplemented with 1 mM ethylenediamine tetraacetic acid (EDTA), followed by five additional washes with the same buffer without EDTA. Washes were completed using a centrifugal filtration device according to the modified protocol of Machczynski et al. (2004). Protein concentration was estimated using Bradford’s assay with bovine serum albumin as the standard at 590 nm (Bradford 1976).

### Sodium dodecyl sulfate-polyacrylamide gel electrophoresis (SDS-PAGE) and zymography

The protein samples were subjected to 10% SDS-PAGE and then incubated with Coomassie Brilliant Blue to enable band visualization. These gels were then bleached using a 10% acetic acid solution (Laemmli 1970) and visualized.

Laccase activity was evaluated by zymography. Proteins were separated using a non-denaturing electrophoretic assay (PAGE 6%) at 4 °C and 100 V, and then incubated in the presence of the chromogenic substrate 2,2-azino-bis (3-ethylbenzothiazoline-6-sulfonic acid) (ABTS) in 100 mM sodium citrate buffer (pH 4.0).

### Enzyme tests and kinetic parameters

Laccase activity was estimated by reading the absorbance values after Lacmeta-mediated oxidation of the ABTS chromogenic substrate (420 nm; ε = 36,000 M^−1^ cm^−1^). Unless otherwise indicated, reactions were performed on microplates with a total reaction volume of 100 µL, comprising 80 µL of 20 mM sodium phosphate buffer, pH 7.0, 10 µL of substrate (1 mM final concentration), and 10 µL of the enzyme (84.72 ng/µL). The reaction was initiated by adding each enzyme to the solution. Measurements were recorded at the desired absorbance at 1 min intervals for 30 min at 37 °C using a MultiScan Go (Thermo Fisher Scientific, Waltham, MA, USA). One unit of laccase activity was defined as the quantity of laccase capable of oxidizing 1 μmol of substrate per minute under the assay conditions. All experiments were performed in triplicate, and the controls were subjected to the same parameters without the addition of the enzyme.

We evaluated the effects of pH changes at 37 °C, in the range of 3.5 to 11.0 using 20 mM of the following buffers: sodium citrate (pH 3.5–6.5), sodium phosphate (pH 6.5–7.0), Tris–HCl (pH 7.0–7.5), HEPES (pH 7.5–8.5), Tris–HCl (pH 8.5–9.0), sodium bicarbonate (pH 9.0–11.0). The optimum temperature was determined by evaluating laccase activity in 20 mM sodium citrate buffer (pH 4.0) at 4 °C, 10 °C, 15 °C, 20 °C, 25 °C, 30 °C, 37 °C, 40 °C, 45 °C, 50 °C, and 60 °C. Thermostability was evaluated by previously incubating the enzyme in sodium citrate buffer, pH 4.0, at temperatures 30 °C, 37 °C, 40 °C, 50 °C and 60 °C. After that, the samples were cooled on ice and subsequently measuring its activity with the substrate.

We then determined the enzyme tolerance in the presence of three detergents, namely, Tween 80, Triton x-100 (nonionic), and SDS (anionic), at a range of concentrations (0–2% (v/v%)). To analyze the effect of salts and metal ions on enzyme activity, we assayed the laccase-mediated oxidation of the ABTS substrate in the presence of CuSO_4_, MgSO_4_, NiCl_2_, CaCl_2_, NaCl, CoCl_2_, KCl, FeSO_4_, MnSO_4_, MnSO_4_, Al(SO_4_)3, and LiSO_4_ at a concentration of 2 mM, using the optimum pH and temperature parameters.

The kinetic parameters k_cat_, K_m_, and V_max_ were evaluated using 0.066 µM enzyme and ABTS substrate between 0.1 and 10 mM under optimal temperature and pH conditions. Kinetic parameters were calculated using nonlinear regression and the Michaelis–Menten equation produced using GraphPad PRISM software (version 9.0) (GraphPad Software, La Jolla, CA, USA).

### Stability assays

The storage stability of LacMeta was evaluated at 4 °C in 20 mM Tris–HCl buffer with 300 mM NaCl (pH 7.5) containing 5% glycerol. The LacMeta samples used in this test were those obtained via gel filtration and evaluated over a 1-year period. Stability was assessed by measuring the respective enzyme activities compared to the newly extracted enzyme at various time points. These residual activity assays were performed in triplicate using 20 mM sodium citrate buffer (pH 4.0) and carried out at 37 °C using 1 mM ABTS as the substrate.

### Dye decoloration

The decoloration capacity of these enzymes was also evaluated. Each test was carried out in 200 µL reaction volumes without stirring in 96-well microplates using phosphate buffer (pH 7.5) and 100 ppm of each dye, with and without ABTS (0.1 mM) as a redox mediator. The reaction was initiated by adding the previously bound copper-purified enzyme and allowed to progress for 48 h at 37 °C. The decoloration of the respective dyes was measured based on the change in absorbance for each dye using a MultiScan Go spectrophotometer (Thermo Fisher Scientific, Waltham, MA, USA) and expressed as a percentage of the original using the following equation, where ‘Ai’ is the initial dye absorbance and ‘At’ is the final absorbance after enzyme treatment. All assays were performed in triplicate.

$${\text{Decoloration }}\left( \% \right) = \left[ {\left( {{\text{Ai}} - {\text{At}}} \right)/{\text{Ai}}} \right]*{1}00$$

### Treatment of water polluting compounds

The decoloration of the malachite green dye was performed at concentrations of 50, 100, 200, 500, 1000, and 2000 mg/L. The tests were performed in triplicate and results expressed in percentage, similar to that for the other dyes mentioned above. For fentin hydroxide, visible UV spectrum tests were performed and a standard curve (0.1–1.0 mg/mL) of the compound was obtained, and the treatments were evaluated according to the linear equation, and the readings were performed using a wavelength of 300 nm. The samples used were previously incubated for 24 h at 37 °C to 150 rpm, and the compound used was at a concentration of 0.6 mg/mL.

### Statistical analysis

Data obtained from comparison standards for pH activity, temperature, detergent treatment, and temporal stability were analyzed using R (4.0.3 version) and subjected to ANOVA analysis and Tukey test, where a probability of less than 5% was considered significant.

## Results

### Sample origin and predicting the domain prediction and identification of the LacMeta enzyme

In silico analyses are important prospecting tools in the annotation of new enzymes, and they can be used to predict the in vitro behavior of these molecules, facilitating their further analysis. The amino acid sequence from LacMeta was subjected to a similarity analysis using the NCBI BLASTp tool with various collections of GenBank/NCBI as the source data. The initial comparisons were made against the “non redundant” (“nr”) database, which revealed that LacMeta shared 80.34% identity with a *Streptomyces rubidus* hypothetical MCO (accession number WP_069465126.1). The second set of evaluations was performed using a manually curated database collection, UniProtKB/SwissProt, and the results indicated that LacMeta shares a maximum identity of only 26.62% with the original characterized laccase from *Fusarium graminearum*. The third set of evaluations was completed to identify more closely related series and used the “Metagenomic proteins” (env_nr) database as the data source. This analysis identified one MCO from one non-cultivable bacterial hydrothermal community (Accession number VAW95009.1) with a sequence identity of 28.03%.

**Fig. 1:**
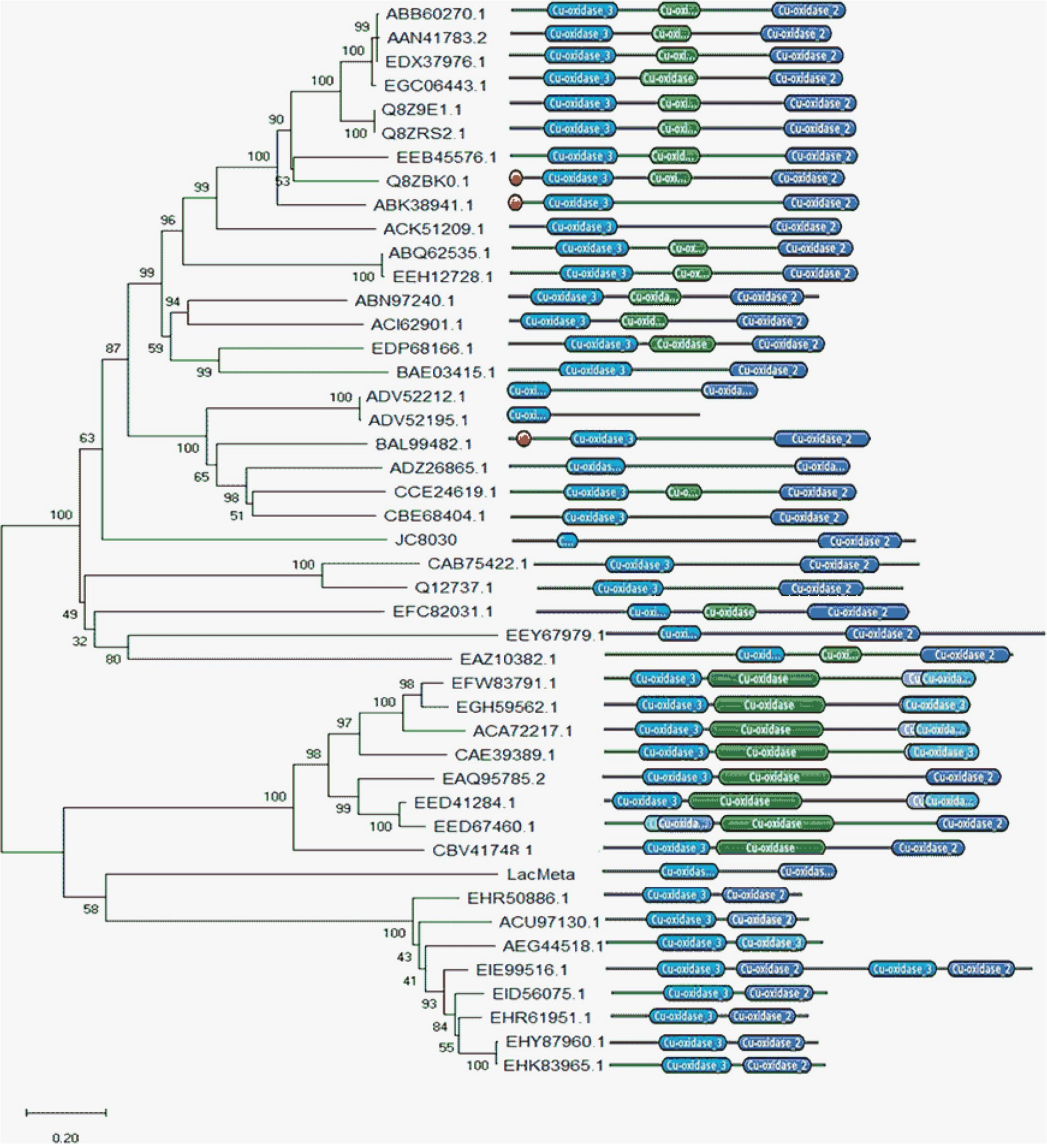
Hierarchical dendrogram obtained for sequences of different families of laccases characterized and deposited in the “Laccase and Multicopper Oxidase Engineering Database,” LccED database, applied by the optimization method for close neighbors (neighbors joining). The domain mapping was followed by the DoMosaics program (Moore et al. 2014)

Using the dedicated “Laccase-characterized enzyme sequences and MCOs” database (“LccED”) as a tool, we performed an in silico functional prediction. This analysis revealed that LacMeta may be a member of the K family, corresponding to the group of “SLAC-like” enzyme—laccase with two domains instead of the traditional three MCO domains. LacMeta shares 41% identity with the conserved domains of *Conexibacter woesei* and 29.15% identity with *Streptomyces xiaopingdaonensis*, both of which belong to the phylum *Actinobacteria*. To better understand the LacMeta enzyme classification, a hierarchical cluster dendrogram was created using DoMosaics and the LccED sequences of I-Bacterial Bilirubin Oxidases, J-Bacterial CueO, H-Bacterial CopA, and K-SLAC-like enzymes (Fig. 1). A total of 44 sequences were analyzed with laccases containing between one and three domains clearly separated into two main subgroups. Branch clustering followed the LccED family nomenclature and LacMeta grouped in the SLAC branch. Within this branch, we identified several enzymes from Actinobacteria, including *Isoptericola variabilis* (AEG44518.1), *Streptomyces viridis* (ACU97130.1), and *Saccharomonospora marina* (EHR5096.1). To obtain more details about the LacMeta sequence, we performed a multiple sequence alignment and showed that the sequences clustered along the three distinct laccase families (I, J, and K, equivalent to bilirubin oxidase, CueO, and SLAC, respectively), and five other sequences also identified in LccED were also included in the K family clusters (Additional file 1: Fig. S1). The alignment of these sequences demonstrated strong conservation of the copper-binding sites and the concatenation ligands within the trinuclear clusters (Additional file 1: Fig. S1). In addition, we noted that the sequences closest to these active sites were most similar in enzymes with similar functions and those belonging to the same family. LacMeta shared the greatest degree of similarity with sequences from the K family, which corroborates the previous predictions for these proteins in our study and those recorded in the LccED (Fig. 1).

### Enzymatic expression and purification

**Fig. 2:**
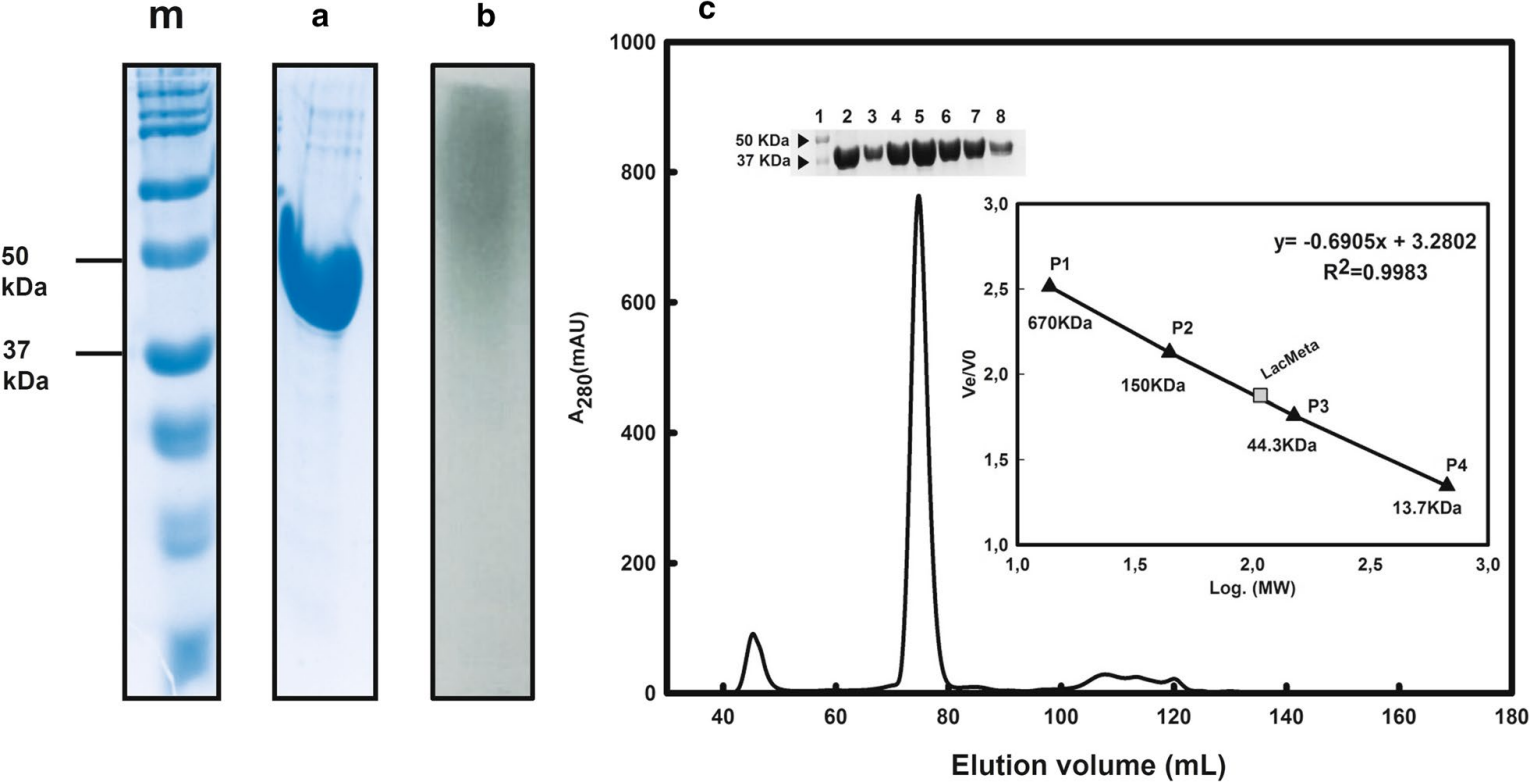
LacMeta expression and purification. m) Molecular mass marker. **a** 10% SDS-PAGE metal affinity purification fraction of the recombinant protein. **b** Native LacMeta gel stained with 1 mM of 2,2′-Azino-bis (3-ethylbenzothiazoline-6-sulfonic acid), ABTS, in sodium citrate buffer pH 4.0. **c** Chromatographic profile of the gel filtration purified metagenomic. The internal image corresponds enzyme equivalent fractions purified laccase (2–8). Molecular mass estimation of LacMeta by gel filtration based on linear correlation. Elution of standard molecular mass of LacMeta and protein standards versus their logarithmic molecular mass values. P1–P4: Proteins used as molecular mass standards: ρ-amino benzoic acid (pABA) (0.13 kDa), Ribonuclease A (13.7 kDa), albumin (43 kDa), (Bovine thyroglobulin (670 kDa), γ-globulin (150 kDa), Protein Standard Mix 15 ± 600 kDa, Sigma, St. Louis, MO, USA)

Optimized expression conditions (30 °C at 200 rpm for 20 h of induction) resulted in enzyme yield of 15.3 mg/L of bacterial cultivation. After overexpression of the protein, the first purification step used the histidine tail-containing recombinant laccase (6× His) and nickel resin Ni–NTA (Qiagen, Venlo, Netherlands) to isolate the LacMeta protein, and these fractions were analyzed by SDS-PAGE (Fig. 2). SDS-PAGE analysis enabled the visualization of a single purified band with a molecular mass between 37 and 50 kDa, which was compatible with the predicted mass (42.74 kDa) for each polypeptide chain in the LacMeta amino acid sequences identified by the ProtParam tool (Fig. 2a). Figure 2B illustrates the results obtained by the non-denaturing electrophoresis assay and shows a band (green) with oxidative activity corresponding to the predicted location of the laccase protein.

Molecular mass exclusion chromatography was used to predict the mass of the LacMeta protein in its native apoenzyme configuration (before the addition of the copper co-factor). Here, we used linear regression analysis of the column void elution volume ratio and the log of the molecular mass at the same elution volume (Ve/Vo) to calculate the size of the apoenzyme. The estimated molecular mass was 107.93 kDa (Fig. 2c), indicating that LacMeta probably adopts a homotrimeric conformation.

### Enzymatic activity and kinetic parameters

**Fig. 3:**
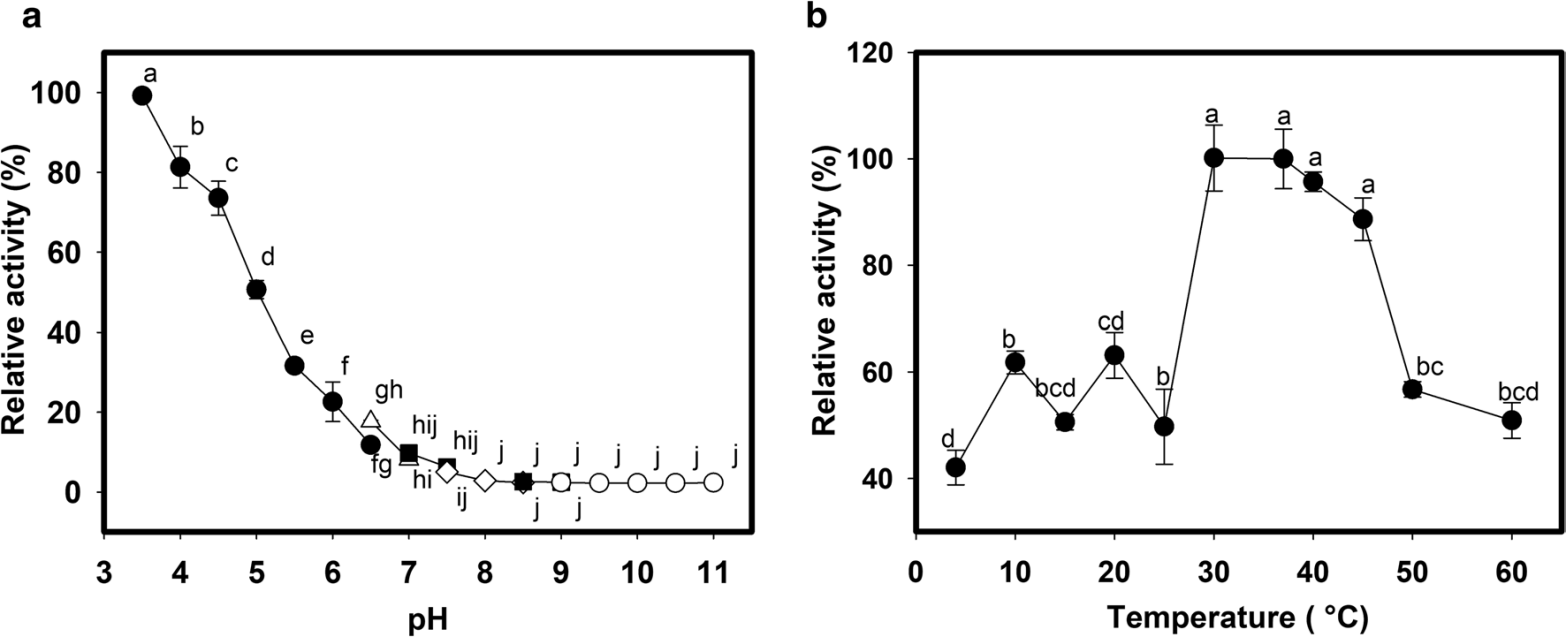
Effects of pH and temperature on LacMeta activity. **a** Effect of pH on laccase activity. The buffers used were: 20 mM sodium citrate (pH 3.5 to 6.5) (black circle), 20 mM sodium phosphate (pH 6.5 to 7.0) (white triangle); 20 mM TRIS–HCl (pH 7.0 to 7.5 and 8.5 to 9.0) (black square), 20 mM HEPES (pH 7.5 to 8.5) (white rhombus) and sodium bicarbonate at 20 mM (9.0 to 11) (white circle). **b** Effect of temperature on activity. An enzymatic activity was measured from 4 to 60 °C in 20 mM sodium citrate buffer (pH 4.0). The tests were performed with 3 replicates. The values followed by the same letter do not differ statistically, according to the ANOVA and Tukey test, with a 5% probability

Our pH evaluations revealed that LacMeta exhibited the most robust enzymatic activity at pH 3.5 (100%) (Fig. 3a), but this low pH resulted in precipitation and denaturation of the enzyme after only 20 min, making this pH impractical for our subsequent evaluations. This means that all subsequent tests were conducted at the second-best activity value (80%, pH 4.0) in 20 mM sodium citrate buffer.

LacMeta activity did not differ significantly between 30 and 45 °C. Thus, we used 37 °C, a common temperature for enzyme assays, for all of the following assessments. In addition, LacMeta only exhibited a loss of relative activity at 4 °C, indicating that this enzyme is extremely thermostable with relevant performance values up to 60 °C (Fig. 3b).

**Fig. 4:**
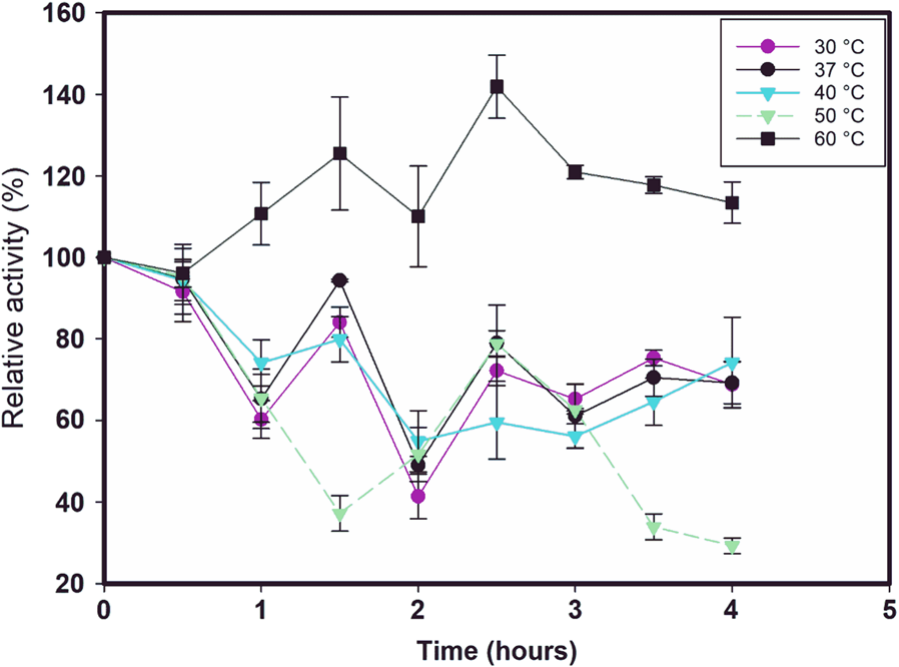
LacMeta thermostability. LacMeta was previously incubated at temperatures 30 °C (purple circle), 37 °C (black circle), 40 °C (blue triangle), 50 °C (green triangle) and 60 °C (black square) Aliquots were removed at different time points for the measurement of residual activity

Interestingly, LacMeta showed high thermostability at 60 °C, reaching 140% relative activity after 2 h and 30 min of pre-incubation, and throughout the whole test (4 h), it maintained at least 100% relative activity. In addition, at 37 °C, only 50% of activity was lost after 2 h, following which the enzyme stabilized at 70% relative activity (Fig. 4).

**Fig. 5:**
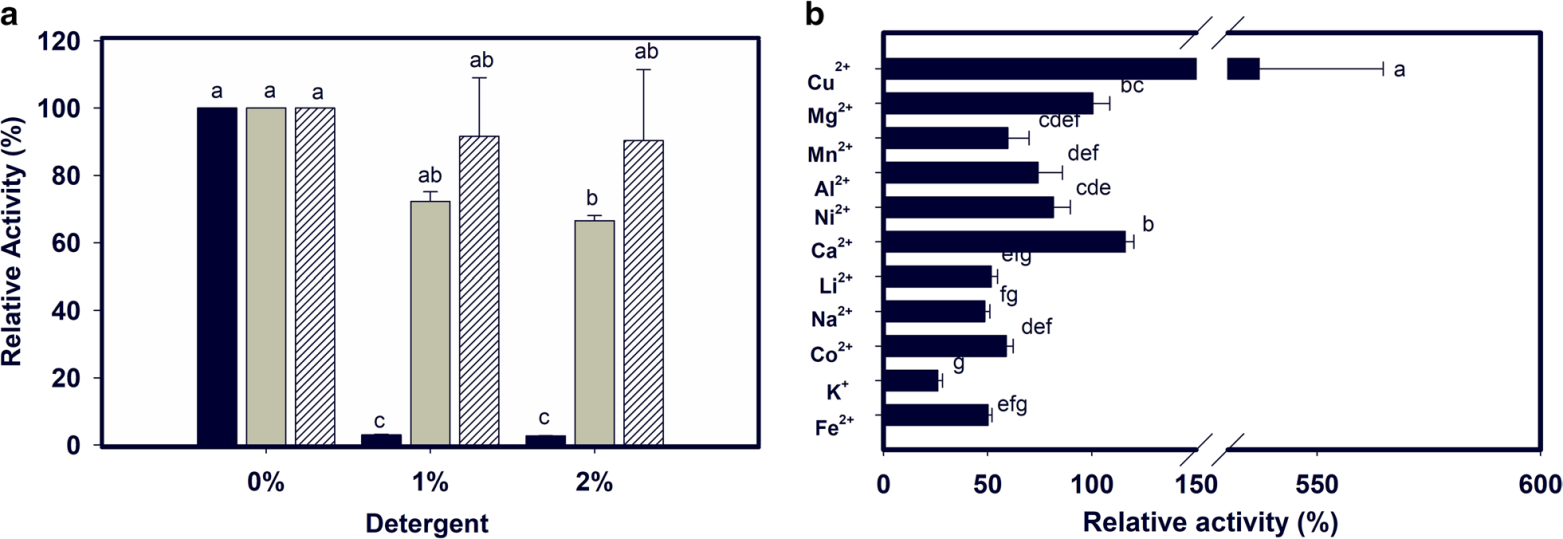
Influence of detergents and ions on laccase enzymatic activity. **a** Reactions were performed using SDS (black bars), Triton x-100 (gray bars) and Tween 80 (striped bars) detergents at concentrations of 0%, 1%, and 2%. **b** Reaction control was performed without the presence of additives (ions) and was used as the basis for the calculation of the other percentages. The tests were performed with 3 replicates. The values followed by the same letter do not differ statistically, according to the ANOVA and Tukey test, with a 5% probability

The effect of detergents on laccase activity was evaluated using previously established parameters (i.e., 37 °C and pH 4.0). When LacMeta was evaluated in the presence of 1–2% Tween 80, it demonstrated some degree of tolerance, maintaining 90% of its residual activity (Fig. 5a).

As expected for multicopper enzymes, LacMeta activity increased considerably in the presence of Cu^2+^ ions (fivefold increase) (Fig. 5b). Potassium ions were shown to exert an inhibitory effect on enzyme activity, with only 26% of the residual activity maintained compared to that of the control. LacMeta was able to tolerate the presence of other cations reasonably well with no cations inducing a loss of more than 50% of residual activity of the enzyme.

Finally, we evaluated the kinetic parameters of this enzyme using the nonphenolic substrate, ABTS, at the optimal temperature and pH. LacMeta obtained K_cat_, K_m_, and V_max_ values of 6.377 ± 0.303 s^−1^, 4.219 mM, and 24.43 μM/min, respectively. When we compared these catalytic parameters with those of other laccases, LacMeta demonstrated catalytic efficiency similar to SLAC (the first reported small laccase (Machczynski et al. 2004), with 1.51 s^−1^ mM^−1^ (Table 1).

**Table 1:** Comparison of Kcat, Km and Kcat/Km with other laccases described in the literature

Protein	Search	Substrate	pH	*Kcat* (s ^−1^)	*Km* (mM)	*Kcat/Km* (s^−1^ mM^−1^)	References
LacMeta	Metagenome	ABTS	4.0	6.337 ± 0.303	4.219 ± 0.3745	1.511	This work
CotA	*Bacillus pumilus*	ABTS	3.6	40.04	0.25394	157.86	Luo et al. (2018)
ThioLacc	*Thioalkalivibrio* sp	ABTS	5.0	2.158	0.0046	0.470	Ausec et al. (2015)
SLAC	*Streptomyces coelicolor*	ABTS	4.0	9.49	5.8	1.611	Prins et al. (2015)
rLac	*Klebsiella pneumoniae*	ABTS	4.0	1.02	0.00533	0.19	Liu et al. (2017)

### Storage stability

**Fig. 6:**
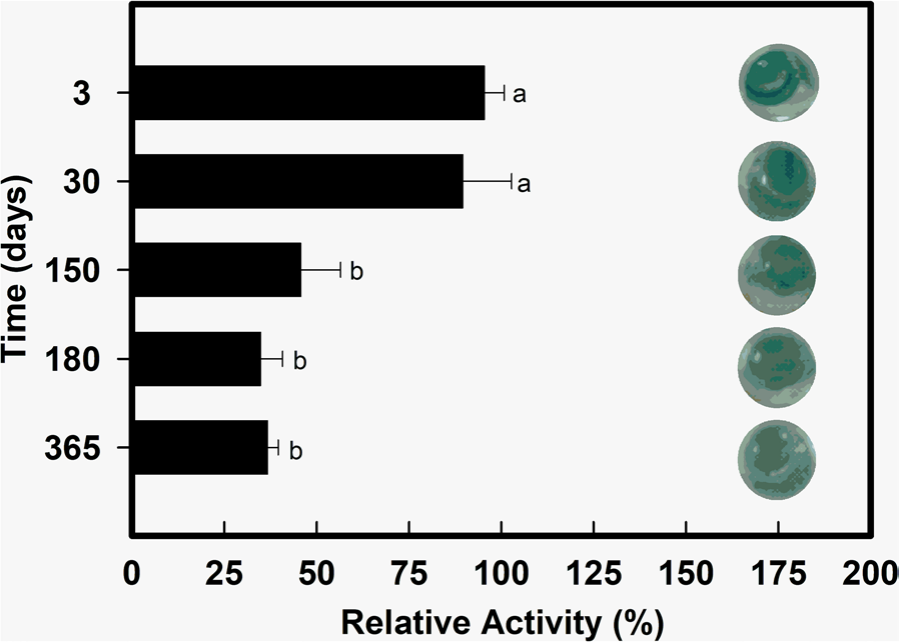
Evaluation of long-term stability. Reactions were performed with purified enzymes after the test, using 2,2′-azino-bis (3-ethylbenzothiazoline-6-sulfonic acid), ABTS, as substrates in the parameters set with optimum enzymatic activity (pH 4.0 and 37 °C). The tests were performed with 3 replicates. The small letters at the top indicate the significant difference between each condition performed in the experiment, according to ANOVA and Tukey test at 5% probability

Stability testing showed that this enzyme was able to retain its activity when stored in a refrigerator at 4 °C for up to 1 year. Activity was evaluated at 3, 30, 150, 180, and 365 days. The final evaluations revealed that gel filtration-purified LacMeta stored at 4 °C retained approximately 34% of its activity after 12 months of storage, and that this residual activity was not significantly less than the residual activity observed at 5 months (Fig. 6).

### Dye decolorization

The ability of LacMeta to degrade various textile dyes was evaluated by observing the decolorization of nine dyes from four different classes with or without a redox mediator (Additional file 1: Table S1). It is interesting to note that when we added 0.1 mM ABTS as a redox mediator, only the bromophenol blue dye maintained its original decoloration value; all the other dyes had an increased rate of decolorization in the presence of ABTS. Methylene blue, trypan blue, and malachite green demonstrated 85%, 83%, and 81% decolorization, respectively, in the presence of the redox mediator. Copete et al. (2015) evaluated the effect of different redox mediators (acetosyringone, syringaldehyde, methyl syringate, and 1-hydroxybenzotriazole) on the decolorization of various dyes, but only syringaldehyde substantially increased the decolorization of Remazol Brilliant Blue R (RBBR) from 2.2 to 56% in their study. These characteristics suggest that this novel laccase could promote bioremediation of various dyes with different chemical structures in the presence of a redox mediator.

### Treatment of water polluting compounds

**Fig. 7:**
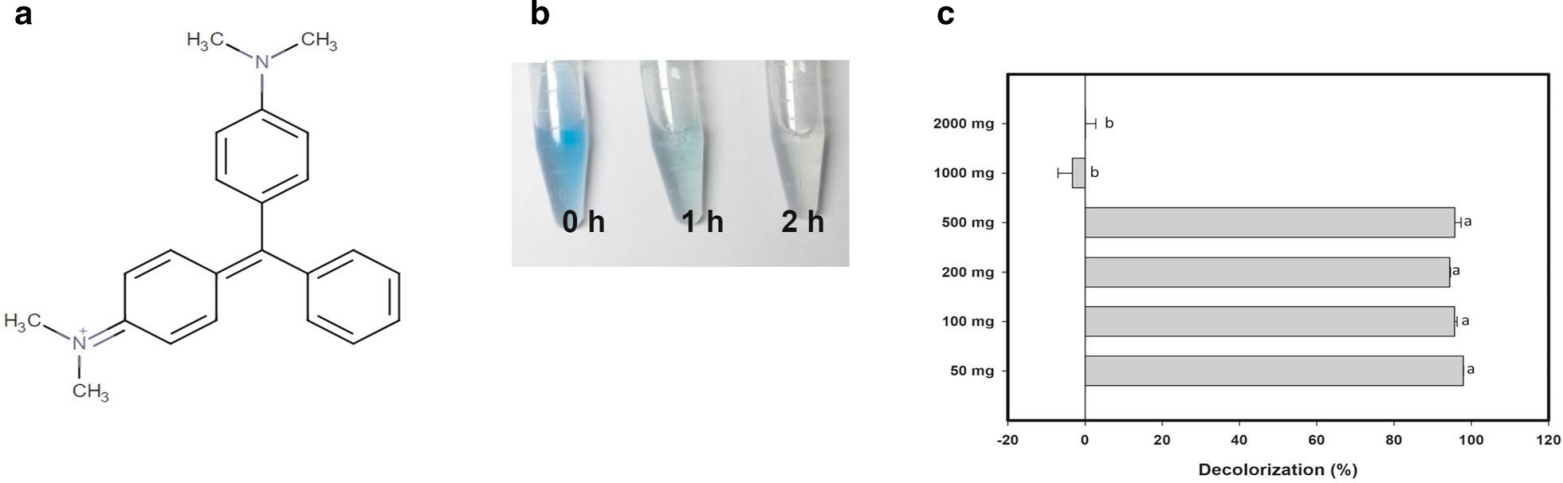
Effect of LacMeta on degradation of Malachite Green. **a** Chemical structure of the dye Malachite Green. **b** Illustration of the decoloration of malachite green when subjected to enzymatic treatment with 0.1 mM ABTS as a redox mediator and stirring (150 rpm) for 2 h. **c** Effect of concentration on Malachite Green percentage of discoloration

Of the results described in this study, the findings regarding the decolorization of malachite green (Fig. 7a), the oldest artificial dye in current use that is structurally related to triphenylmethane, are probably the most interesting. LacMeta showed a high affinity for and ability to degrade this dye; in particular, when subjected to agitation, it was able to produce 100% decoloration in a very short time (2 h) (Fig. 7b, c).

**Fig. 8:**
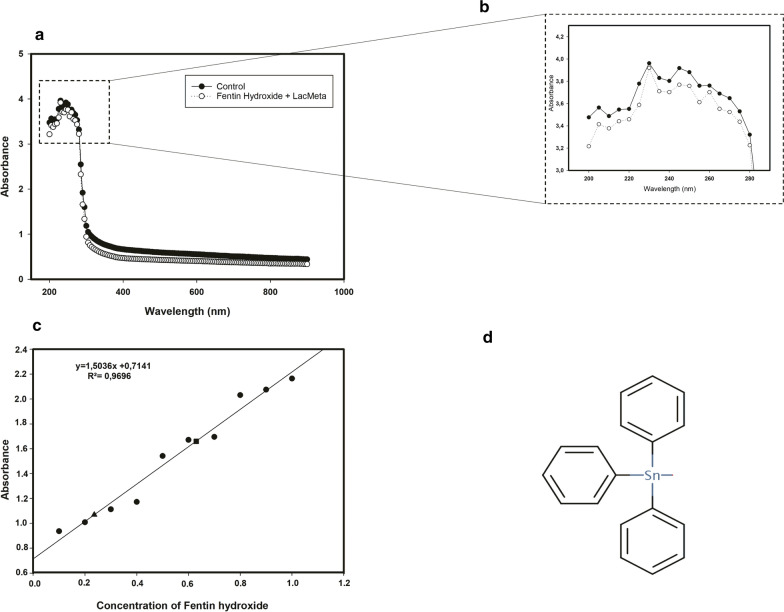
Effect of LacMeta on degradation fentin hydroxide. **a** UV–Vis spectra of 200–900 nm. **b** UV–Vis spectra of 200–280 nm. **c** Degradation of fentin hydroxide (300 nm). Black triangle: initial concentration; Black square: final concentration after LacMeta treatment. **d** Chemical structure of fentin hydroxide. Tests were performed for 24 h at 37 °C to 150 rpm

Fentin hydroxide (triphenyltin hydroxide) is a pesticide with antifungal activity that is widely used in cotton and bean crops in Brazil (Fig. 8d). According to the standard curve, LacMeta was able to degrade more than half of the compound, from 0.6 to 0.2 mg/mL (Fig. 8c), which was also confirmed by reading the absorption peaks that decreased after 24 h of treatment (Fig. 8a).

## Discussion

This work was successful in the cloning and enzymatic characterization of a new two-domain laccase, obtained from the eucalyptus soil metagenome. Molecular mass exclusion chromatography indicated that LacMeta probably has a homotrimeric structure, this is consistent with our predictions that suggest that LacMeta is a two-domain bacterial laccase sharing some similarity with previously characterized SLAC enzymes, given the fact that most characterized SLACs adopt a homotrimer conformation (Olbrich et al. 2019). There are, however, some SLACs that do not conform to this norm, one example of which is the oligomeric *Streptomyces anulatus* laccase, which adopts an estimated eight-subunit structure (Lisov et al. 2019). These laccases are believed to have evolved into three domains through the duplication of domains in older single-domain proteins (Nakamura et al. 2003).

LacMeta also showed 50% residual activity at pH 5.0, which decreased gradually as the pH increased. These results suggest that this enzyme prefers acidic environments when metabolizing ABTS as a substrate. This preference for acidic pH in the presence of ABTS has also been described for several *Streptomyces* sp. laccases, including *Streptomyces* sp. SB086, which has an optimal pH between 4.0 and 5.0 (Fernandes et al. 2014). Incidentally, a group of SLACs that have garnered attention because their enzymatic activity spans a wide pH range, which can vary from 3.0 to 9.0, depending on the substrate (oxidase enzymes depend on different redox potentials to act on different groups). The pH range is a critical parameter for industrial applications, especially for enzymatic activity in the presence of phenolic compounds used in the textile industry (Dubé et al. 2008). For temperature, only at 50 °C did LacMeta have substantial losses of activity; after 4 h of testing, the relative activity was 30%, and even at 2 h and 30 min, and 3 h, a part of the activity was recovered. Optimum temperatures for laccase activity and thermostability may vary greatly between species, and this variety could be even more diverse compared to that demonstrated by this enzyme in other genera (Madhavi and Lele 2009). Similar results regarding the optimal temperature have been reported for *Streptomyces* sp. C1, which has been shown to have an optimum temperature of 40 °C (Lu et al. 2013), while rLac has an optimal temperature of 35 °C (Liu et al. 2017). However, other *Streptomyces* spp. SLACs have been described as having optimal temperature of 50 °C and have been reported to lose 50% of their activity below 30 °C (Fernandes et al. 2014). By comparing LacMeta and rLac, we were able to show that rLac loses up to 90% of its activity at 60 °C, while LacMeta maintains 50% of its residual activity at this temperature.

SDS completely inhibited the activity of LacMeta, which is similar to what happened in the other evaluations of metagenomic laccase (Qu et al. 2018), which also demonstrated a loss of enzyme activity at 1% SDS. Enzyme activity was also stimulated by Ca^2+^, which increased the relative activity to 115%. The relative activity of SLAC of *Streptomyces* sp. C1 has also been reported to be increased by calcium to 145% (Nakamura et al. 2003).

In addition, this enzyme was shown to be an efficient decolorizing agent for both methylene blue and crystal violet, without requiring any redox mediator. The ability of rLac to degrade dyes, including Congo red, crystal violet, and malachite green, without a redox mediator did not exceed 27% in a previous study (Li et al. 2020). LacMeta’s ability to degrade these compounds, even in the absence of a redox mediator, is a strong indication that its performance may be robust enough to facilitate textile effluent treatment. Malachite green dye is widely used as an ectoparasiticide, fungicide, food dye, food additive, medical disinfectant, and industrial dye (Barapatre et al. 2017). However, there are a number of concerns regarding its use, as it has been linked to adverse immune and reproductive responses and is known to possess some potential carcinogenic effects (Srivastava et al. 2004).

Fentin hydroxide belongs to the group of organotin compounds, which have a broad spectrum of activity as biocides in general, and are also known to cause immunotoxicity, endocrine disruption, and changes in the reproduction rate of animals (Grote et al. 2006; Sarpa et al. 2010). Specific studies on bioremediation of fentin hydroxide little studied. A proposal for tributytin, another organotin group compound, bioremediation was carried out using *Enterobacter cloacae*; however, it did not yield satisfactory results, and the concentration of the compound decreased; though, the authors indicated that this process must be carried out together with other forms of treatment (Sakultantimetha et al. 2011). These results demonstrate the importance of identifying new biomolecules that can be used for the bioremediation of this and other similar compounds.

LacMeta is a new small metagenomic laccase, which can be an important feature while the industry is markedly lacking in new raw material to carry out new biotechnological processes, since the vast majority of enzymes used in the industry derive from a restricted number of isolated microorganisms that often represent only 1% of the entire environmental microbiome. LacMeta showed maximum activity at acidic pH (ABTS) and maintained its activity across a broad range of temperatures. In addition, LacMeta retained its activity even after prolonged storage and was shown to rapidly degrade seven different textile dyes from four different classes and a fungicide. This suggests that this enzyme may be ideal for industrial applications, wastewater bioremediation and warrants a closer evaluation in industrial settings.

## Supplementary Information

**Additional file 1**: Multisequence Alignment for LacMeta against LccED Sequences and LacMeta Performance on Dye decoloration.

## Data Availability

All data generated or analysed during this study are included in this published article (and its additional files). Sequence data that support the findings of this study have been deposited in GenBank with the primary accession codes MN974156. Requests for material should be made to the corresponding author.
