# Supplementary material for: Broad thermal spectrum metagenomic laccase with action for dye decolorization and fentin hydroxide treatment
Source: AMB Express. 2022 Mar 23;12:38. doi: 10.1186/s13568-022-01375-0 (PMC8943092; doi:10.1186/s13568-022-01375-0)
Supplement: Supplementary file 1 — Additional file 1: Multisequence Alignment for LacMeta against LccED Sequences and LacMeta Performance on Dye decoloration. [file 13568_2022_1375_MOESM1_ESM.pdf]

Additional File 1: Multisequence Alignment for LacMeta against LccED Sequences and LacMeta Performance on Dye decoloration

AMB Express

Broad thermal spectrum metagenomic laccase with action for dye decolorization and fentin hydroxide treatment

Natália Sarmanho Monteiro Lima[a,b,c #], Elisângela Soares Gomes-Pepe[a,b #], João Carlos Campanharo [a], Eliana Gertrudes de Macedo Lemos[a,b *]

[a] Department of Agricultural and Environmental Biotechnology, São Paulo State University, Jaboticabal Campus, São Paulo State, Brazil.), 14884-900, Jaboticabal, São Paulo State, Brazil.

[b] Molecular Biology Laboratory, Institute for Research in Bioenergy (IPBEN), 14884-900, Jaboticabal, São Paulo State, Brazil.

[c] Graduate Program in Agricultural and Livestock Microbiology, São Paulo State University (UNESP), School of Agricultural and Veterinarian Sciences, Jaboticabal, SP, Brazil

[#] These authors contributed equally to this manuscript.

*Corresponding author: eliana.lemos@unesp.br

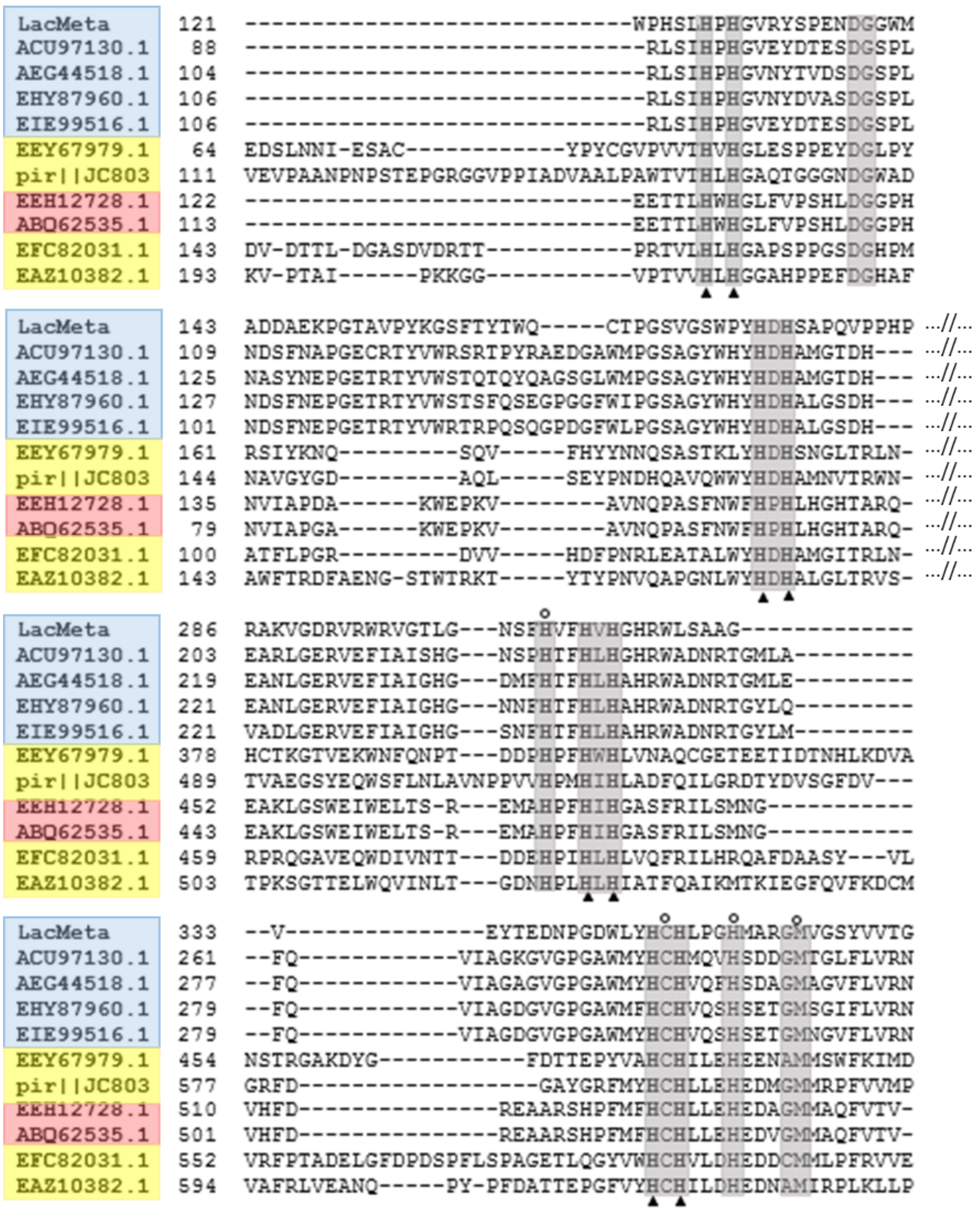

Fig. S1. Multiple sequence alignment obtained for sequences of different families of laccases characterized and deposited in the “Laccase and Multicopper Oxidase Engineering Database”, LccED database, applied Clustal Omega version 1.0.3 (Sievers et al. 2014).

Table S1. LacMeta effect on decoloration of synthetic dyes at 100 ppm

**Dyes**		**Chemical class**	**λmax (nm)**	**No mediator**		**With mediator**	
				**% of decoloration****			
RBBR*	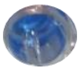	Anthraquinone	595	2%±0.01^{f}	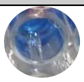	28%±0.06 ^{e}	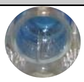
Methylene Blue	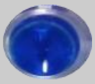	Thiazide	666	47%±0.71^{a}	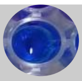	85%±0.002^{cd}	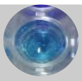
Malachite Green	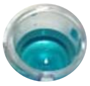	Triphenylmethane	618	1%±0.06^{f}	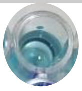	81%±0.005^{a}	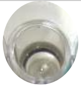
Trypan Blue	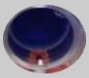	Dyazo	607	12%±0.05^{f}	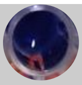	83%±0.005^{a}	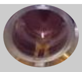
Phenol Red	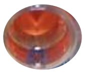	Triphenylmethane	558	7%±0.04^{f}	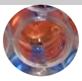	31%±0.09^{de}	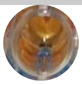
Bromophenol Blue	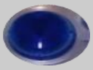	Triphenylmethane	580	1%±0.04^{f}	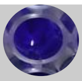	1%±0.02^{f}	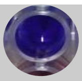
Congo Red	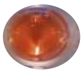	Dyazo	510	2%±0.03^{f}	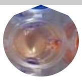	68%±0.003^{ab}	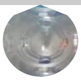
Crystal Violet	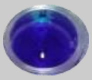	Triphenylmethane	583	52%±0.31^{bc}	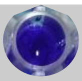	56%±0.01^{bc}	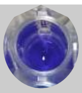
Bromocresol Green	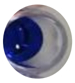	Triphenylmethane	620	1%±0.15^{f}	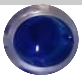	3%±0.07^{f}	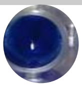

*Remazol Brillante Blue R

** Values in lines followed by the same letters are not significantly different, $P < 0.05$
